# Identifying Priorities, Targets, and Actions for the Long-term Social and Ecological Management of Invasive Non-Native Species

**DOI:** 10.1007/s00267-021-01541-3

**Published:** 2021-09-29

**Authors:** Pablo García-Díaz, Lía Montti, Priscila Ana Powell, Euan Phimister, José Cristóbal Pizarro, Laura Fasola, Bárbara Langdon, Aníbal Pauchard, Eduardo Raffo, Joselyn Bastías, Gabriella Damasceno, Alessandra Fidelis, Magdalena F. Huerta, Eirini Linardaki, Jaime Moyano, Martín A. Núñez, María Ignacia Ortiz, Ignacio Rodríguez-Jorquera, Ignacio Roesler, Jorge A. Tomasevic, David F. R. P. Burslem, Mário Cava, Xavier Lambin

**Affiliations:** 1 School of Biological Sciences, University of Aberdeen, Aberdeen, AB24 2TZ UK; 2 Instituto de Investigaciones Marinas y Costeras (IIMyC), FCEyN-Universidad Nacional de Mar del Plata-CONICET, CC 1260, 7600 Mar del Plata, Argentina; 3 Instituto de Geología de Costas y del Cuaternario (IGCyC), FCEyN-Universidad Nacional de Mar del Plata-CIC, Funes 3350, 7600 Mar del Plata, Argentina; 4 Instituto de Ecología Regional (IER, UNT, CONICET) and Facultad de Ciencias Naturales e IMl, UNT, Residencia Universitaria de Horco Molle, Yerba Buena, Tucumán, Argentina; 5 Business School, University of Aberdeen, Aberdeen, AB24 3QY UK; 6 Business School, University of Stellenbosch, PO Box 610, Bellville, 7535 South Africa; 7 Laboratorio de Estudios del Antropoceno (LEA), Facultad de Ciencias Forestales, Universidad de Concepción, Concepción, Chile; 8 Consejo Nacional de Investigaciones Científicas y Técnicas (CONICET)-Dirección Regional Patagonia Norte de la Administración de Parques Nacionales, O’Connor 1188, 8400-San Carlos de Bariloche, Río Negro, Argentina; 9 Laboratorio de Invasiones Biológicas (LIB), Facultad de Ciencias Forestales, Universidad de Concepción, Concepción, Chile; 10 Institute of Ecology and Biodiversity (IEB), Santiago, Chile; 11 Servicio Agrícola y Ganadero, Gobierno de Chile, Valdivia, Chile; 12 Lab of Vegetation Ecology, Instituto de Biociências, Universidade Estadual Paulista (UNESP), Av. 24A, Rio Claro, 13506-900 Brazil; 13 Centro de Humedales Río Cruces (CEHUM), Universidad Austral de Chile, Valdivia, Chile; 14 Grupo de Ecología de Invasiones, INIBIOMA, CONICET, Universidad Nacional del Comahue, Quintral 1250, San Carlos de Bariloche, CP 8400 Argentina; 15 Department of Biology and Biochemistry, University of Houston, Houston, TX 77204 USA; 16 Programa Patagonia, Departamento de Conservación de Aves Argentinas/Asociación Ornitológica del Plata, Buenos Aires, C1249 AAB Argentina; 17 Departamento de Análisis de Sistemas Complejos, Fundación Bariloche, CONICET, Av. Bustillo 9400, San Carlos de Bariloche, CP 8400 Argentina; 18 EDGE of Existence-Zoological Society of London, London, UK

**Keywords:** Alien species, Collaborative process, Expert knowledge, Latin America, Natural resource management planning, Uncertainty

## Abstract

Formulating effective management plans for addressing the impacts of invasive non-native species (INNS) requires the definition of clear priorities and tangible targets, and the recognition of the plurality of societal values assigned to these species. These tasks require a multi-disciplinary approach and the involvement of stakeholders. Here, we describe procedures to integrate multiple sources of information to formulate management priorities, targets, and high-level actions for the management of INNS. We follow five good-practice criteria: justified, evidence-informed, actionable, quantifiable, and flexible. We used expert knowledge methods to compile 17 lists of ecological, social, and economic impacts of lodgepole pines (*Pinus contorta*) and American mink (*Neovison vison*) in Chile and Argentina, the privet (*Ligustrum lucidum*) in Argentina, the yellow-jacket wasp (*Vespula germanica*) in Chile, and grasses (*Urochloa brizantha* and *Urochloa decumbens*) in Brazil. INNS plants caused a greater number of impacts than INNS animals, although more socio-economic impacts were listed for INNS animals than for plants. These impacts were ranked according to their magnitude and level of confidence on the information used for the ranking to prioritise impacts and assign them one of four high-level actions—do nothing, monitor, research, and immediate active management. We showed that it is possible to formulate management priorities, targets, and high-level actions for a variety of INNS and with variable levels of available information. This is vital in a world where the problems caused by INNS continue to increase, and there is a parallel growth in the implementation of management plans to deal with them.

## Introduction

**Fig. 1:**
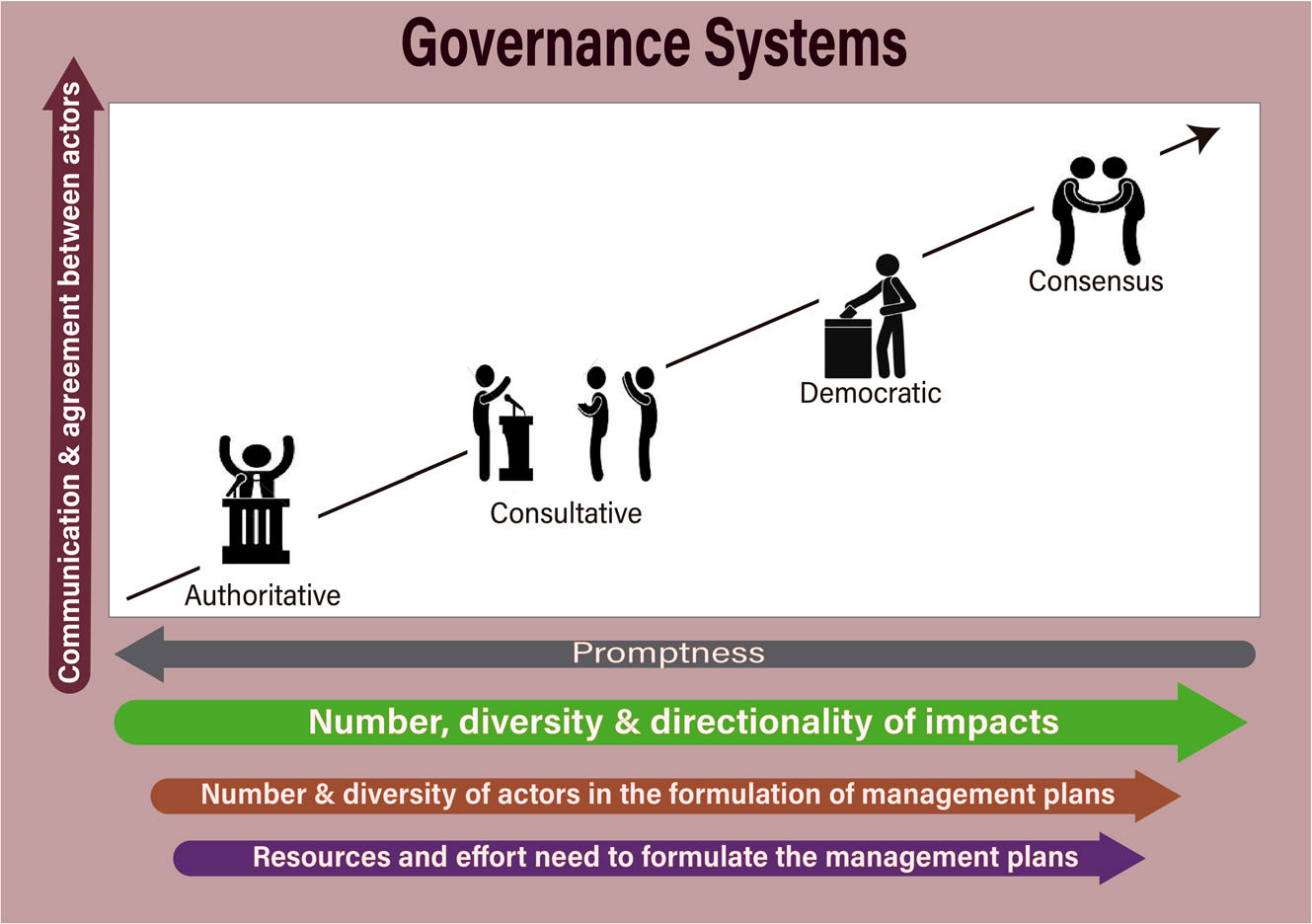
Decision-making rules in four broad types of the governance system to formulate INNS management plans (modified from the categories in Conroy and Peterson 2013). The level of participation and agreement between actors increases from left to right. Increased participation levels are generally more suited for formulating management plans in situations with numerous and varied impacts and actors involved. However, this comes at the expense of promptness (swiftness with which a plan can be formulated) and with an associated increased need for resources and effort. Our methods and individual tools can be adapted to any of these four systems, although they tend to be more appropriate for collaborative approaches

Invasive non-native species (INNS, hereafter; also referenced as invasive alien species) are those non-native species that have established and spread outside their native ranges (Blackburn et al. 2011). Some of them cause severe social, economic, cultural, and environmental impacts, affecting human livelihoods, biodiversity, and ecosystem services (Blackburn et al. 2019; Linders et al. 2020; Pyšek et al. 2020). The impacts and costs arising from these harmful INNS are predicted to rise over the next decades worldwide (Essl et al. 2020b; Seebens et al. 2021; Diagne et al. 2021). Therefore, it is not a surprise that managing INNS ranks high among the priorities of a broad array of governmental, non-governmental and private institutions, organisations, and agencies worldwide (Turbelin et al. 2016; Convention on Biological Diversity 2020; Essl et al. 2020a; Hulme 2021). The eradication of many of these INNS is currently unfeasible, and instead, long-term plans are needed for sustained management to address their impacts (Bomford and O’Brien 1995; Green and Grosholz 2021; Robertson et al. 2020; García-Díaz et al. 2021). The nature and development of these management plans are highly context-dependent (Fig. 1), but there is a shared need for formulating clear priorities and measurable targets to evaluate whether they are effective.

The identification of priorities and specification of targets for the management of INNS requires collaboration among disciplines and the explicit incorporation of diverse societal perspectives to ensure that management plans meet the varied expectations and values of society and stakeholders (Crowley et al. 2017a; Vaz et al. 2017; Kapitza et al. 2019). This approach is essential when the goal is to generate and sustain a commitment to management in perpetuity (Bridger et al. 2019). The need for transdisciplinary approaches to tackle complex socio-ecological issues has long been recognised by fields such as sustainability science (Lang et al. 2012; Mattor et al. 2014), and our work aligns well with this literature and principles in terms of collaborative research and team-building actions for gaining a common understanding of our research problem.

The degree to which this collaboration and integration of perspectives are achieved is variable and depends on a wide array of factors (Fig. 1). A key reason to include multiple views is that different sectors of society commonly place different values on the same INNS, including contrasting values (Estévez et al. 2015; Beever et al. 2019; Kapitza et al. 2019; Oficialdegui et al. 2020). This divergence of values may arise, in some instances, because an INNS may have negative environmental impacts, but positive social or economic benefits (Andriantsoa et al. 2020; Vimercati et al. 2020). Spatial and temporal spillover effects are particularly relevant. For instance, a private company may reap benefits from planting and harvesting non-native pines (*Pinus* spp.), but this can be to the detriment of society if the trees spread beyond the plantations and invade natural protected areas with high cultural and ecological significance or productive pastures (Nuñez et al. 2017, 2021; Bravo-Vargas et al. 2019). Failing to recognise the multiplicity of values that emerge from the variety of positive and negative in situ and spillover INNS impacts is bound to create controversies and conflicts that yield subpar management plans (Estévez et al. 2015; Crowley et al. 2017a, 2017b; Beever et al. 2019).

There is a need for approaches that facilitate collaboration among a variety of actors to identify management priorities and uncertainties in an inclusive and structured way. These approaches explicitly permit and encourage disagreement, providing means to resolve these disagreements and translate them into concrete and feasible actions (Novoa et al. 2018; Van Woensel 2019; Clement 2020; Evans 2021). We define participants as actors interested in, affected by, or with a stake in the management of the focal INNS, including but not limited to decision-makers, managers, experts, and representatives of the affected communities and companies. The extent and level of engagement of participants will depend on the governance arrangements (Fig. 1), where agreeing on a common language and decision-making procedures from the outset are fundamental to the success of the more collaborative and inclusive approaches (Craig 2018; Van Woensel 2019).

**Fig. 2:**
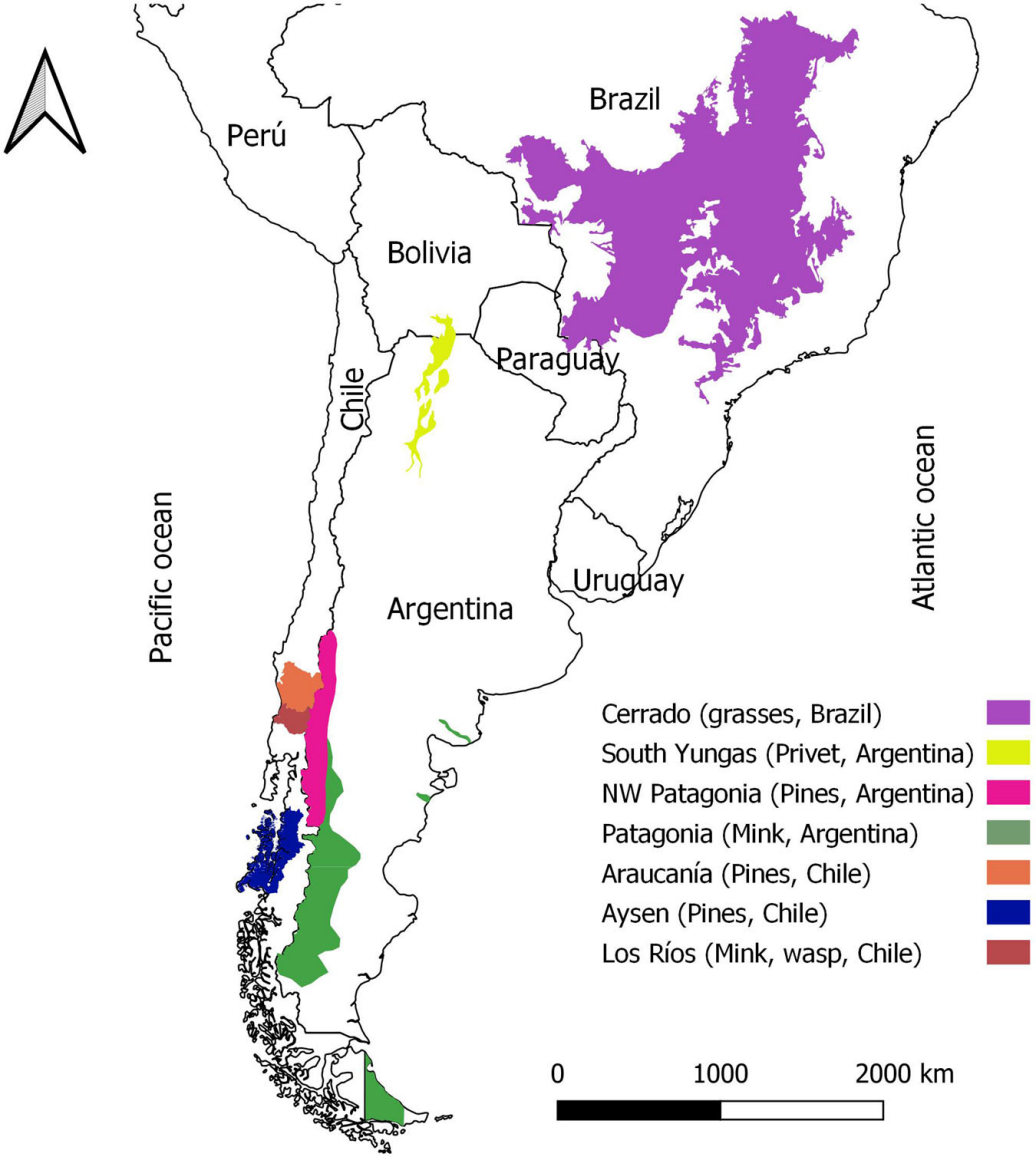
Geographical location of the seven case studies in Latin America. Note that the sections coloured correspond to the areas where the plan will be applicable, which are case-specific and can range from biomes to administrative jurisdictions

In 2019, we commenced a multinational programme aimed at advancing the management of harmful INNS in Latin America (Fig. 2). Our group includes ecologists, economists, social scientists, and practitioners from Argentina, Brazil, Chile, and the United Kingdom (Lambin et al. 2020). At the start of our programme, we encountered difficulties in defining management priorities and targets, and linking and integrating the different backgrounds and disciplines of the participants. There was confusion about the goals of managing our target INNS and instances of conflation of management goals and means to achieve those goals (e.g. mitigation of impacts versus population control). In addition, there was a greater emphasis on ecological than social and economic impacts, which led to underestimating the impacts of INNS and hindered the valuation of the costs caused by INNS. Also, there was an overt focus on negative INNS impacts with a poor consideration of positive impacts, even though understanding both types is vital. Furthermore, there was a general sense that the scarcity of information was a severe barrier to formulating objectives and actions.

Here, we showcase a collaborative approach to formulating priorities, targets, and high-level actions that is based on the experiences and lessons we gained to overcome the obstacles in our programme (Online Resource 2). We focus exclusively on formulating priorities, targets, and high-level actions; the design and implementation of specific activities to meet those priorities and targets are beyond the scope of this work (see further guidance in the “Discussion”). Our experience illustrates the difficulties that INNS management programmes face across the world, particularly in areas where INNS only recently have been considered a significant problem. Therefore, we expect that our procedures will be especially useful for actors involved in the management of INNS who are beginning to craft their plans and are facing difficulties similar to ours.

In the following sections, we describe and detail the rationale and procedures for each of the three stages of our prioritisation and target-setting exercise (Online Resource 2). We illustrate our procedures with seven case studies of invasive plants (four examples) and animals (three examples) in Latin America. These were (Fig. 2) lodggepole pine (*Pinus contorta*) in the Araucanía and Aysén Regions (Chile) and in the Northwestern Patagonia Region (Argentina), privet (*Ligustrum lucidum*) in the South Yungas Forest (Argentina), invasive grasses (*Urochloa brizantha* and *Urochloa decumbens*) in the Cerrado (Brazil), American mink (*Neovison vison*) in Austral Patagonia (Argentina) and the Los Ríos Region (Chile), and yellow-jacket wasps (*Vespula germanica*) in the Los Ríos Region (Chile). The size of the areas covered by each case study differed (4,877 to 2,000,000 km^2^), reflecting the region over which their respective management plans could be envisaged or implemented. They ranged from entire biomes (e.g. temperate rainforests and wetlands in Los Ríos, Chile) to well-delimited administrative jurisdictions, reflecting the multiscale nature of INNS issues. The simultaneous application of our process to a variety of case studies; geographic and political scales (three countries); social, economic, and human density scenarios; and taxonomic scope is an attempt to demonstrate the applicability of our approach across a wide array of potential circumstances. We conclude by discussing potential extensions, future directions, and limitations of our process.

### Defining the Scope and Aims of Long-term Inns Management Plans

We started by recognising that, when the target INNS cannot be eradicated, management plans should focus on addressing their negative and positive impacts (Lodge et al. 2016; Dunham et al. 2020; García-Díaz et al. 2021). Unfortunately, the complexity and ambiguity often associated with impact assessments and management can limit the definition of priorities and targets to manage INNS impacts effectively. First, there are multiple ways of investigating and measuring impacts, compounded by the fact that social and economic impacts often depend on the values and perceptions of the stakeholders (Ricciardi et al. 2013; Beever et al. 2019; Kapitza et al. 2019). This produces ambiguity because the management of INNS impacts can be approached from alternate perspectives. In addition, there is scant information on the impacts of many INNS, even when they are intensely managed (Simberloff 2003; Latombe et al. 2019; Crystal-Ornelas and Lockwood 2020a, 2020b). This adds uncertainty that can become a major barrier to the ideal notion of implementing management plans to mitigate INNS impacts.

Our procedures were intended to aid actors involved in INNS management activities in navigating these challenges and arriving at impact outcomes and assets that should be a strategic priority, and high-level actions to deal with them. We incorporated the different dimensions of impact defined by the International Union for the Conservation of Nature (IUCN) Invasive Species Specialist Group and used them in standardised assessments of INNS impact magnitude (see https://www.iucn.org/theme/species/our-work/invasive-species/eicat and http://www.iucngisd.org/gisd/howto.php). These are the impact outcomes (the consequences of the impact), their magnitude and direction (positive or negative), and the mechanisms underpinning those outcomes. We added another dimension that was relevant to our case, the species, or assets impacted (assets hereafter for simplicity; an asset is any type of resource).

Five key tenets of well-designed public policy and natural resource management planning should underpin long-term INNS management plans (Groves and Game 2016; Dunn 2017; Evans 2021). First, a management plan should be *justifiable*. This criterion addresses the demand and motivation for designing a plan and springing into action. Second, the plans should be *evidence-informed*, which is fulfilled by making use of the best available evidence that is relevant to the purported methods and goals (Russell-Smith et al. 2015; Rose et al. 2018; Van Woensel 2019). Third, the plan should be made *actionable* by defining, at least in broad terms, the actions to be undertaken. It should also be possible to measure the targets of the plan to shape actions and monitor progress, leading to the fourth criterion—the presence of *quantifiable targets*. Fifth, there should be *flexibility* so the management plan can be revised to account for new information and deal with novel and unforeseen situations (adaptive management).

Adhering to these five criteria will also improve accountability and transparency in the production and implementation of a management plan. We acknowledge that the management of INNS is complex and multifaceted, and it is likely that there will be instances in which trade-offs among criteria will be unavoidable. For example, achieving an appropriate level of flexibility may come at the cost of a detailed quantification of progress towards the targets. Resolving these trade-offs will require further analyses to evaluate which solutions achieve the most effective management outcomes. Guidance and criteria for assessing and ranking more detailed activities than those defined by the third criterion can be found elsewhere (Groves and Game 2016; Green and Grosholz 2021; Mill et al. 2020; Dunham et al. 2020; García-Díaz et al. 2021).

## Methods

### From Impact Outcomes and Assets to High-level Plans: A Systematic Sequential Approach

Our procedures integrate the five criteria via three sequential stages that help formulate priorities, targets, and high-level actions for management plans while allowing for the consideration of varied perspectives (Online Resource 2). The focus on impact outcomes by design means that the rationale for the management plan is explicit (justifiable). In our first stage, participants draw on existing information (evidence-informed) to inventory and assess INNS impacts semi-quantitatively and qualitatively. The information collated ideally will include the magnitude and direction (either positive or negative) of all possible outcomes of the impacts of INNS on all possible assets, and mechanisms will be attributed to each of those impact outcomes. This stage results in a catalogue of combinations of impact outcomes by assets by impact mechanism. Our second stage assists the participants in prioritising those impact outcomes on the basis of their magnitude and uncertainty (Game et al. 2013), and serves to classify each impact outcome in one of four high-level action categories (actionable): do nothing, monitor, research, and immediate active management. Once high-level actions have been identified for each impact outcome and asset, the participants define quantitative indicators to measure those impact outcomes in real life (third stage). These indicators can be used both to improve the understanding of the magnitude of the impacts (reducing uncertainties; flexible) and to track the progress of the management plan (quantifiable).

#### First stage: inventory and evaluating impacts

Our first stage builds on the standardised lists and methods used by the IUCN to inventory and assesses the impacts of INNS. We relied on the standardised lists of impact outcomes, impact mechanisms, and the methods to quantify the magnitude and uncertainty of impacts underpinning the Environmental Impact Classification of Alien Taxa (EICAT; Blackburn et al. 2014; Hawkins et al. 2015). Although we drew on these pre-existing tools, we did not use the same protocols, nor was it our objective to do so (Kumschick et al. 2020). Indeed, EICAT is centred on environmental impacts and it is commonly applied to estimate a single magnitude of the effects of each INNS, which corresponds to the highest possible impact magnitude supported by the evidence (Blackburn et al. 2014; Hawkins et al. 2015; Kumschick et al. 2020).

Our protocol casts a broader net and includes not only realised but also potential impacts on the basis of a review of information and experiences obtained outside our case study areas. In addition, we emphasised individual impact outcomes and assets impacted rather than coming up with a single category for each INNS. The standardised lists of impact outcomes and mechanisms include environmental (biotic and abiotic) and social and economic impacts, which allows for a collective assessment across these categories. The EICAT categories and criteria (data deficient, minimal concern, minor, moderate, major, and massive) were applied to each combination of impact outcome by asset by impact mechanism. This allowed us to gather information on the magnitude and uncertainty of the impact outcomes by asset and by mechanism level rather than at the species level. Adapting tested standardised methods means that the methods are sound and comparable with other work that applied these methods and that our approach could be transferable beyond our case examples (Kumschick et al. 2017, 2020).

For each of our seven case examples, we contacted two to four experts by email and requested that they complete a comprehensive impact score spreadsheet (see Online Resource 1 and data availability statement for further details). Expert knowledge has proven useful in informing INNS management, particularly when drawn from interdisciplinary teams that have the capacity to integrate disparate evidence sources and weight uncertainties (Roy et al. 2020). All the experts (*n* = 16) were part of our research programme and are co-authors of this paper. Our protocols readily can be extended to include other participants, including other experts and stakeholders. The experts’ affiliations included universities, non-governmental organisations, and government agencies. The impact score spreadsheet included eight columns (impact outcome, impact mechanism, maximum impact [EICAT classification level], level of confidence, species or asset impacted, direction of the impact [positive or negative], comments and other details, and references and supporting information) plus additional personal and context-specific details (see Online Resource 1 and Data availability statement for further details). The last column supported meeting the evidence-informed criterion.

At this stage, the experts were asked to fill in the impact score spreadsheet with as much detail as possible, including both realised and potential impact outcomes. Potential impacts were automatically classified as data deficient and low level of confidence. Potential impacts are usually excluded from EICAT evaluations (Kumschick et al. 2020), but we included them because they are fundamental for monitoring and awareness of plausible and likely current or future impacts (actionable). Consistent with existing protocols for using expert knowledge (Hemming et al. 2018), each expert was contacted individually and independently and provided with an individual impact score spreadsheet. Experts were instructed to complete the task within 2 weeks and to try to take no more than 2 h to fulfil the task. Upon returning their impact score spreadsheets, experts were instructed to share and discuss their spreadsheets with other experts within the same case study. After this exercise, individual experts had the opportunity to revise their spreadsheets and return their updated versions.

The last step was another round of revisions in which external experts not involved in the previous steps joined in a facilitated discussion (Online Resource 2). This activity was intended to encourage the experts to think outside their own systems and provide a potentially challenging outsider view. In our case, this last step took place during a facilitated workshop held in San Carlos de Bariloche (Argentina; 3–4 December 2019), where all the participant experts and three researchers from the University of Aberdeen and Universidade Estadual Paulista who were not involved in the previous steps worked in three groups covering pines in Chile and Argentina, American mink in Chile and Argentina and yellow-jacket wasps in Chile, and privet in Argentina and invasive grasses in Brazil. The impact score spreadsheets were openly shared and discussed, and the experts revised them as necessary. The spreadsheets for each of the seven case examples were merged for subsequent analysis.

#### Second stage: prioritising impacts and defining high-level actions

The wealth of information generated in the first stage established a solid evidence basis for formulating management plans (evidence-informed). However, the information needed to be translated in a way that could lead to meaningful actions (actionable). Tackling all of the impact outcomes simultaneously is not feasible. Therefore, in the second stage, we used the information on impact outcomes, assets impacted, and magnitudes and associated levels of confidence to narrow down the list and select the most pressing impact outcomes that needed to be addressed immediately. In other words, we defined the impact outcomes that will be prioritised for management.

Once a priority list is ready, it is possible to decide on an appropriate course of action for each selected impact outcome and asset. Here, the magnitude (EICAT category) and the level of confidence (uncertainty) play a key role. The impact outcomes are aligned along two axes on the basis of the approach described by Game et al. (2013). The *x*-axis indicates the magnitude of the impact outcome and the *y*-axis represents the level of confidence. The plot is then divided into four domains identifying the high-level actions to undertake for each priority impact outcome (actionable): do nothing, monitor, immediate active management, and research. This is a pragmatic way of summarising the importance of each impact outcome and guiding the formulation of high-level strategic responses while accounting for the uncertainties in the information available (level of confidence) (also see Milner-Gulland and Shea 2017; Rowland et al. 2014).

**Fig. 3:**
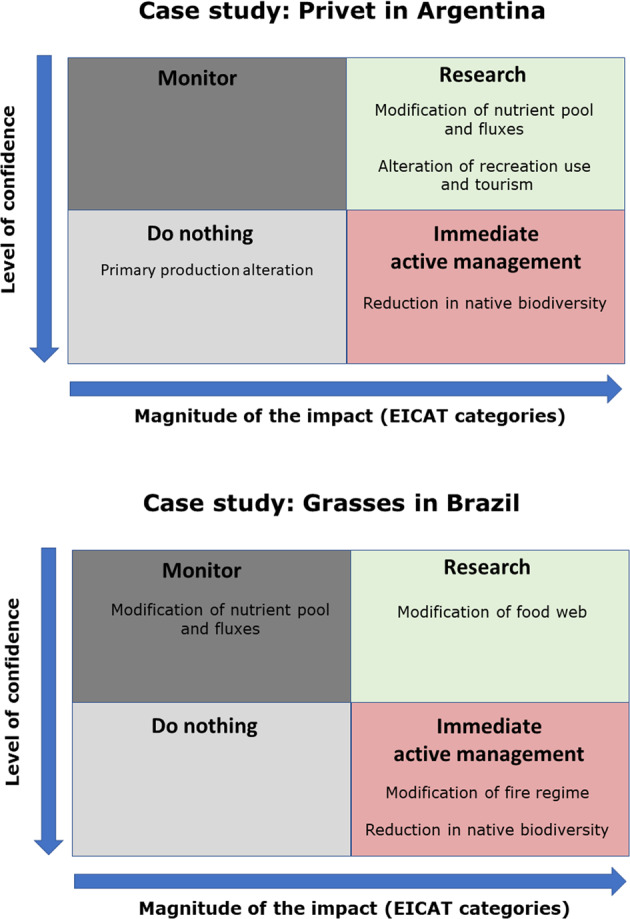
Two examples of prioritising and assigning high-level actions to impact outcomes based on their Environmental Impact Classification of Alien Taxa magnitude and level of confidence

During the workshop, the experts discussed and identified in conversation with peers and external experts the impact outcomes that should be taken into account in a management plan for their case studies given the information in their spreadsheets. We highlight the impact outcomes assigned to immediate active management, but provide two representative examples of the full classification (Fig. 3). The impact outcomes that require monitoring or additional research should still be included because the new information obtained on those impact outcomes should be used to update the spreadsheets and, in time, revise the management priorities and targets (flexible). These impacts can be assigned to either research or monitoring on the basis of the magnitude and level of confidence (Fig. 3). This is aligned with the well-known principles of adaptive management of natural resources and INNS (Foxcroft and McGeoch 2011; Westgate et al. 2013; Lambin et al. 2020).

#### Third stage: quantitative measures of impact outcomes and assets impacted

Prescribing quantitative indicators is essential to diagnosing the extent and degree of the impact outcomes and to monitoring the progress and success of the management plan (quantifiable). No single quantitative indicator fits all cases, and therefore we did not use a standardised list or approach to guide the process. In addition, the number of quantitative indicators does not need to match the number of priority impact outcomes. Multiple indicators can be used to quantify one impact outcome, and in some instances, a single indicator might measure several impact outcomes.

### Preliminary Assessment of the Potential Usefulness of the Procedures and their Likelihood of Adoption

We conducted a preliminary assessment of the potential usefulness, caveats, and avenues for improvement of our procedures through an informal, six-question survey of our network of colleagues in Latin America. Our network of informal contacts included 55 experts affiliated with government agencies (60.5%), scientific organisations (30.9%), and environmental non-governmental organisations (3.6%) from Argentina (72.7%), Chile (23.6%), and Uruguay (3.6%). The experts had experience in INNS, conservation, and natural resource management. The informal survey in English and Spanish is available from Online Resource 1.

## Results

### First Stage: Inventory and Evaluating Impacts

We obtained 17 impact score spreadsheets (one of the experts evaluated two species), which in the aggregate listed 206 unique impact outcomes by impact mechanisms by assets across the seven case examples (mean and standard deviation by case example 29.7 ± 12.2, range: 17–46). The number of impact outcomes per spreadsheet was higher for invasive plants than animals (Online Resource 3), and there was no apparent relationship between the spatial extent of each case study or the time experts took to complete their spreadsheets and the number of impact outcomes they listed (Online Resource 3).

**Fig. 4:**
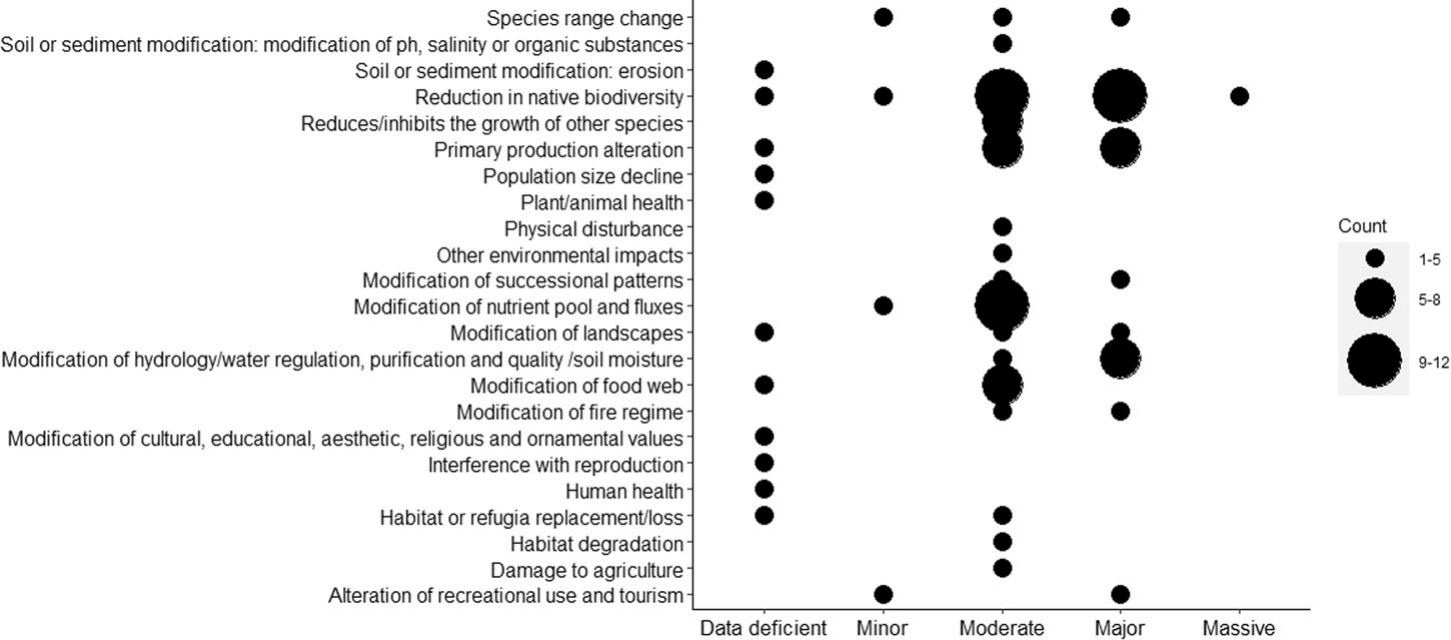
Summary of the impacts of plant INNS in four case studies in Latin America. Impacts are organised by their Environmental Impact Classification of Alien Taxa impact category (*x*-axis) and impact outcome (*y*-axis)

**Fig. 5:**
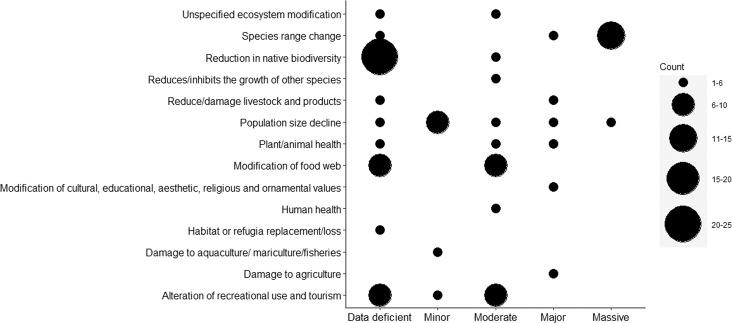
Summary of the impacts of animal INNS in three case studies in Latin America. Impacts are organised by their Environmental Impact Classification of Alien Taxa impact category (*x*-axis) and impact outcome (*y*-axis)

A total of 172 assets were listed as impacted by our case study INNS. Across the seven case studies, the five most prevalent impact outcomes included three environmental and two social and economic types (Figs. 4 and 5): reduction in native biodiversity (environmental; 47 cases; 22.8%), alteration of recreational use and tourism (social and economic; 14 cases; 6.8%), decline in native population size (environmental; 13 cases; 6.3%), damage to agriculture (social and economic; 10 cases; 4.9%), and modification of food webs (environmental; 10 cases; 4.9%). The five most prevalent impact mechanisms corresponded to chemical, physical, or structural impacts on ecosystems (66 cases; 32.0%), predation (62 cases; 30.0%), competition (42 cases; 20.4%), interaction with other invasive species (11 cases; 5.3%), and grazing, herbivory, or browsing (9 cases; 4.4%). The majority of impact outcomes were assessed as negative (187; 90.8%). The magnitude of the impact outcomes (EICAT categories) varied widely across the seven case studies, even when assessing the same INNS in different locations (Online Resource 4). Ranked by magnitude, there were 12 cases of massive impact outcomes (5.8%), 49 of major impact outcomes (23.8%), 70 moderate (34.0%), 18 minor (8.7%), and 10 minimal concern impact outcomes (4.9%). A further 47 impact outcomes were considered to be data deficient (22.8%). The impacts of plant INNS tended to be more severe (major and massive) than those of animal INNS (Online Resource 4). See Online Resource 1 and Data availability statement for summaries and the aggregated Impact score spreadsheet.

### Second Stage: Prioritising Impacts and Defining High-level Actions

Across our seven case examples, only 18 of the 206 impact outcomes (8.7%) were selected for immediate active management (Table 1) (percentage by case example, mean ± standard deviation: 12.2 ± 10.2%; range: 2.2–31.3%). All of the impact outcomes selected for immediate active management were negative; 10 were environmental (55.6%), and eight were social and economic (44.4%).

**Table 1:** Impact outcomes prioritised for immediate active management and quantitative measures chosen for seven case examples in Latin America

Case study	Impact outcomes chosen for immediate active management	Quantitative measures of impact outcomes^a^
Pines in Chile	1. Modification of fire regime 2. Damage to agriculture 3. Modification of hydrology/water regulation, purification and quality/soil moisture 4. Reduction in native biodiversity 5. Alteration of recreational use and tourism	1. Fuel load [impact outcome: 1] 2. Biomass/primary productivity of grass [impact outcomes: 2; 4] 3. Hydrological (flow) model [impact outcome: 3] 4. Species richness and presence of INNS [impact outcomes: 2; 4; 5] 5. Human preferences for different landscapes [impact outcomes: 4; 5]
Pines in Argentina	1. Modification of fire regime 2. Damage to agriculture 3. Modification of hydrology/water regulation, purification and quality/soil moisture	1. Fuel load [impact outcome: 1] 2. Biomass/primary productivity of grass [impact outcome: 2] 3. Hydrological (flow) model [impact outcome: 3]
Privet in Argentina	1. Reduction in native biodiversity	1. Woody plant richness and abundance [impact outcome: 1]
Grasses in Brazil	1. Reduction in biodiversity 2. Modification of fire regimes	1. Plant richness and abundance [impact outcomes: 1] 2. Biomass of invasive grasses [impact outcomes: 2]
American mink in Argentina	1. Population size decline 2. Modification of cultural, educational, aesthetic, religious and ornamental values	1. Breeding success of affected native birds [impact outcomes: 1; 2] 2. Population trends of affected native birds [impact outcomes: 1; 2]
American mink in Chile	1. Population size decline 2. Damage to agriculture 3. Modification of cultural, educational, aesthetic, religious and ornamental values	1. Proportion of households that change their production due to attacks [impact outcomes: 2; 3] 2. Production losses due to attacks [impact outcomes: 2; 3] 3. Breeding success of affected native birds [impact outcomes: 1; 3] 4. Population trends of affected native birds [impact outcomes: 1; 3]
Yellow-jacket wasps in Chile	1. Damage to agriculture 2. Alteration of recreational use and tourism	1. Proportion of beehives lost to yellow jackets [impact outcome: 1] 2. Decline in the number of tourists visiting the area [impact outcome: 2]

The lists of priority impact outcomes were based on the magnitude (IUCN’s Environmental Impact Classification of Alien Taxa—EICAT category) and the uncertainty about those impacts. See Fig. 2 for details on the geographical location and scope of each case study

^a^Text in square brackets link the quantitative measures to specific impact outcomes listed in the adjacent box to the left

### Third Stage: Quantitative Measures of Impact Outcomes and Assets Impacted

The experts identified two to five quantitative indicators to measure impact outcomes and assets impacted for their respective case studies (see examples in Table 1). The indicators were specific to each case study and experts recorded as many indicators as necessary. If management actions include population control, the number of individuals removed and the resulting reduction in abundance should always be quantified and recorded, along with estimates of uncertainty around these measures.

### Preliminary Assessment of the Potential Usefulness of the Procedures and their Likelihood of Adoption

Of the 55 colleagues we contacted, 33 responded to our informal survey. These 33 respondents were affiliated with government agencies (57.6%), scientific organisations (33.3%), and environmental non-governmental organisations (9.1%) from Argentina (57.0%), Chile (39.4%), and Uruguay (6.1%). Of those 33, 91% considered our process interesting and 66.7% as a novel or partially novel (question 1); 84.8% understood and could follow the three stages easily (question 2). To improve our approach (question 3), the participants suggested including information about current and potential invasion risk (area occupied by the target INNS), social perceptions, economic impacts and how to quantify them, and assessments of the costs, benefits, and feasibility of different actions. Despite these recommendations for improving our approach, 60% indicated that the collaborative workflow and methods could be useful for them. The classification of each impact outcome into four high-level action categories was mentioned as particularly useful for selecting and applying actions by some respondents (question 4). Seventy per cent of respondents agreed that the management of INNS should focus on impacts rather than on the species itself (question 5). Only three (9.1%) respondents considered our proposal to focus on impacts as inadequate or not useful, and the remaining 21.2% did not provide an opinion.

When asked about what would be needed for the adoption of our procedures (question 6), the respondents mentioned training on how to use the procedures and the need for better capacity of interdisciplinary work to incorporate varied views into INNS management plans. In addition, there was general agreement that this type of process and the long-term INNS management can fail without sufficient economic resources to support their implementation (e.g. salaries). The latter concern is common among environmental management programmes rather than specific to our procedures.

Our non-systematic approach to gathering informed opinions about our procedures served as a first evaluation and a source of criticism designed to help clarify the purpose and methods of our approach as presented here. We recognise that it might be biased towards positive responses, although the feedback highlighted above contains both positive and critical feedback. As examples of changes induced by these responses, we have emphasised that it is essential to consider positive INNS impacts when crafting management plans. Likewise, the recommendations to include invasion risk and cost–benefit analyses prompted us to explain that, although vital, those belong to the design and implementation of activities phase instead of the formulation of priorities and targets for management plans.

## Discussion

A complex network of actors, values, priorities, and vested interests characterises the context in which long-term management plans for INNS need to operate, yet we believe that these challenging circumstances should not preclude or preempt the development of effective plans to address the pressing issue of INNS impacts (Woodford et al. 2016; Latombe et al. 2019; Probert et al. 2020). We have presented and illustrated an approach and workflow that builds on pre-existing and accepted tools for formulating priorities, targets, and high-level actions for the long-term management of INNS in a systematic, transparent, inclusive, and standardised way. Our approach meets five central criteria for good public policy and natural resource management (to ensure they are justifiable, evidence-informed, actionable, quantifiable, and flexible), indicating that it is possible to contend with the problem of INNS while following good practice and responding in a timely fashion. The fact that only 8.7% of the impact outcomes initially identified were chosen for immediate active management illustrates that it is possible to reduce the number of target impact outcomes to a practical number. This swiftness is an advantage given that the management plans usually are formulated over a restricted time frame such as those arising from windows of opportunity.

The logical next step once priorities and targets are ready will be to define instruments, policies, and activities to implement the high-level actions contained in the management plan (Braysher 2017; Green and Grosholz 2021; García-Díaz et al. 2021). That is beyond the remit of our manuscript, and we refer readers to Conroy and Peterson (2013), Groves and Game (2016), Braysher (2017), and García-Díaz et al. (2021) for further guidance. Nevertheless, multi-criteria decision analysis (Liu et al. 2011; Davies et al. 2013; Adem Esmail and Geneletti 2018) is a potentially suitable option given that it is likely that more than one impact outcome would be the target of the plans—this was the case in six out of our seven examples (Table 1). Value of information analysis and simulation modelling can be useful for designing activities for tackling impact outcomes within the research and monitoring categories (Bennett et al. 2018; Cullinane Thomas et al. 2019; Bertolino et al. 2020). Regardless of the tools employed, it is fundamental that the lessons learned and information obtained during implementation are used to revise the priorities, targets, and actions, starting with the impact score spreadsheets, to verify that they remain current and fit-for-purpose (criterion 5: flexible).

There are limitations and caveats to our procedures and case studies. For instance, a broader range of actors, stakeholders, and perspectives should be incorporated in all the stages (Crowley et al. 2017a; Samson et al. 2017; Novoa et al. 2018; Kapitza et al. 2019; Shackleton et al. 2019). This will ensure that a management plan is representative of the perspectives, impact outcomes, impact directions, and assets impacted by the INNS. Our seven case examples were intended as demonstrative, and therefore we did not emphasise the inclusion of a broader set of stakeholders. Ideally, INNS management plans will be co-developed with stakeholders and managers (e.g. Novoa et al. 2018; Clement 2020; Evans 2021; O’Connor et al. 2021). Stakeholder analyses, focus groups, consultation, and even community assemblies can play a role in incorporating diverse perspectives (Novoa et al. 2018; Nyumba et al. 2018; Sutherland et al. 2018). Furthermore, drawing on research from the field of sustainability can be a productive venue to find transdisciplinary frameworks that can be adapted to the participatory management of INNS, as real-world problems require the engagement of a multitude of stakeholders and scientists from various disciplines (Lang et al. 2012; Mattor et al. 2014). The suitability of these approaches will depend on the purpose and scope of the plan, and time, resource, and policy constraints (Fig. 1).

The involvement of a plurality of actors is not always possible and depends on the existing governance and relevant decision-making arrangements (Fig. 1). In addition, resource limitations could severely hinder the ability to consult and cooperate with stakeholders and other relevant groups (Fig. 1), as highlighted by the respondents to our informal survey. Managing expectations and power differentials and ensuring the inclusion of relevant actors is crucial to participatory processes (Frumento et al. 2019). Facilitators can manage the procedures described here to address potential issues of representation and power relations. For example, anonymising the responses before sharing with other actors and experts, facilitating discussions, and the use of formal techniques such as “serious games” in which some actors take on the role of other actors to understand their perspectives can help deal with these issues (Madani et al. 2017; Frumento et al. 2019). Although our procedures are more suited for collaborative processes, it is possible to adapt them for many governance arrangements (Fig. 1). For example, a selected group of autocratic decision-makers in an agency can still use our procedures for eliciting priorities and targets. Likewise, a consultative or advisory process can be guided through our procedures.

Our procedures, and particularly our use of EICAT procedures for assessing the magnitude of impacts, might not cover all social and economic impacts. Our procedures can be extended to include the recently developed social and economic impacts of alien taxa (SEICAT) methods (Bacher et al. 2018), or other existing social and economic assessment techniques to complement the use of EICAT (Martinez-Cillero et al. 2019; Linders et al. 2020; Milanović et al. 2020). The inclusion of a wide range of stakeholders, as mentioned before, will also help guarantee the adequate representation of social and economic impacts. However, although EICAT has been endorsed by the IUCN (https://www.iucn.org/theme/species/our-work/invasive-species/eicat), SEICAT has not, and alternative classification methods exist (Kumschick et al. 2012; Nentwig et al. 2016; Martinez-Cillero et al. 2019). In addition, the impact outcomes prioritised for immediate active management across our seven case studies were evenly distributed between environmental and social and economic categories, suggesting that our procedures were not necessarily biased against or toward either of those two types. The inclusion of qualitative indicators, such as changes in reported attitudes and well-being of stakeholders, would be a welcome addition to the measurement of impacts outcomes and impacted assets (Kapitza et al. 2019).

We applied our methods to formulate priorities, targets, and high-level actions for addressing the impacts of individual INNS. It also is possible to use our procedures to develop plans for managing impacts across multiple INNS given that a particular set of impact outcomes and assets affected can be common among species (e.g. alteration of fire regimes; see Table 1). Nevertheless, in many circumstances, individual INNS have already been singled out, for example through priority lists, and there is a need for management plans tailored to those INNS (Roy et al. 2014; McGeoch et al. 2015; Carboneras et al. 2017). Selecting priority INNS is beyond the remit of our approach. There are many approaches for prioritising individual INNS on the basis of risk or strategic foresight (Roy et al. 2014; Lodge et al. 2016; Probert et al. 2020).

We have presented and demonstrated our procedures as a stand-alone method. This is not a prerequisite, and instead, it can be conceptualised as a toolbox for formulating priorities, defining targets, and scoping high-level actions for the long-term management of INNS. Elements of our techniques can be used to inform and complement plans developed with other approaches. For example, our methods can be embedded within the theory of change models, which depict how to progress from the current situation to the desired objective of the management plan (Allen et al. 2017; Robertson et al. 2020). We are confident that the use of our methods and techniques will prove useful in untangling the complex task of managing INNS over long time horizons, and can support organisations that seek to promote the development of INNS management plans.

## Supplementary information

ESM 1

ESM 2

ESM 3

ESM 4

## Data Availability

Materials and anonymised summary data can be found in the Online Resource 1 and will also be made publicly available from the UK Environmental Information Data Centre (10.5285/d00a647a-16ec-4d2a-a3a3-ad59597e8ca2) and FigShare (10.6084/m9.figshare.15134418).

## References

[CR1] Adem Esmail B, Geneletti D (2018). Multi-criteria decision analysis for nature conservation: a review of 20 years of applications.. Methods Ecol Evol.

[CR2] Allen W, Cruz J, Warburton B (2017). How decision support systems can benefit from a Theory of Change approach.. Environ Manag.

[CR3] Andriantsoa R, Jones J, Achimescu V (2020). Perceived socio-economic impacts of the marbled crayfish invasion in Madagascar.. PLoS ONE.

[CR4] Bacher S, Blackburn TM, Essl F (2018). Socio-economic impact classification of alien taxa (SEICAT).. Methods Ecol Evol.

[CR5] Beever EA, Simberloff D, Crowley SL (2019). Social-ecological mismatches create conservation challenges in introduced species management.. Front Ecol Environ.

[CR6] Bennett JR, Maxwell SL, Martin AE (2018). When to monitor and when to act: value of information theory for multiple management units and limited budgets.. J Appl Ecol.

[CR7] Bertolino S, Sciandra C, Bosso L (2020). Spatially explicit models as tools for implementing effective management strategies for invasive alien mammals.. Mammal Rev.

[CR8] Blackburn TM, Pyšek P, Bacher S (2011). A proposed unified framework for biological invasions.. Trends Ecol Evol.

[CR9] Blackburn TM, Essl F, Evans T (2014). A unified classification of alien species based on the magnitude of their environmental impacts.. PLoS Biol.

[CR10] Blackburn TM, Bellard C, Ricciardi A (2019). Alien versus native species as drivers of recent extinctions.. Front Ecol Environ.

[CR11] Bomford M, O’Brien P (1995). Eradication or control for vertebrate pests?. Wildl Soc Bull.

[CR12] Bravo-Vargas V, García RA, Pizarro JC, Pauchard A (2019). Do people care about pine invasions? Visitor perceptions and willingness to pay for pine control in a protected area.. J Environ Manag.

[CR13] Braysher M (2017) Managing Australia’s pest animals. CSIRO, Canberra

[CR14] Bridger JC, Alter TR, Frumento PZ, Howard TM, Adams LB (2019) Community engagement theory for a new natural resource management paradigm. In: Martin P, Alter TR, Hine D, Howard T (eds) Community-based control of invasive species. CSIRO-CABI, Clayton South, VIC; Wallingford, UK, Boston, p 84–96

[CR15] Carboneras C, Genovesi P, Vilà M (2017). A prioritised list of invasive alien species to assist the effective implementation of EU legislation.. J Appl Ecol.

[CR16] Clement S (2020) Governing the anthropocene: novel ecosystems, transformation and environmental policy. Springer, Cham, Switzerland

[CR17] Conroy MJ, Peterson JT (2013). Decision making in natural resource management: a structured, adaptive approach.

[CR18] Convention on Biological Diversity (2020) Zero draft of the post-2020 Global Biodiversity Framework. https://www.cbd.int/doc/c/efb0/1f84/a892b98d2982a829962b6371/wg2020-02-03-en.pdf. Accessed 19 Mar 2020

[CR19] Craig C (2018) How does government listen to scientists? Springer, Cham, Switzerland

[CR20] Crowley SL, Hinchliffe S, McDonald RA (2017). Invasive species management will benefit from social impact assessment.. J Appl Ecol.

[CR21] Crowley SL, Hinchliffe S, McDonald RA (2017). Conflict in invasive species management.. Front Ecol Environ.

[CR22] Crystal-Ornelas R, Lockwood JL (2020). Cumulative meta-analysis identifies declining but negative impacts of invasive species on richness after 20 yr. Ecology.

[CR23] Crystal-Ornelas R, Lockwood JL (2020). The ‘known unknowns’ of invasive species impact measurement.. Biol Invasions.

[CR24] Cullinane Thomas CM, Sofaer HR, Cline SA, Jarnevich CS (2019). Integrating landscape simulation models with economic and decision tools for invasive species control.. Manag Biol Invasions.

[CR25] Davies AL, Bryce R, Redpath SM (2013). Use of multicriteria decision analysis to address conservation conflicts.. Conserv Biol.

[CR26] Diagne C, Leroy B, Vaissière A-C (2021). High and rising economic costs of biological invasions worldwide.. Nature.

[CR27] Dunham JB, Arismendi I, Murphy C (2020). What to do when invaders are out of control?. WIREs Water.

[CR28] Dunn WN (2017) Public policy analysis: an integrated approach. Routledge, NY, USA

[CR29] Essl F, Lenzner B, Bacher S (2020). Drivers of future alien species impacts: an expert-based assessment.. Glob Change Biol.

[CR30] Essl F, Latombe G, Lenzner B (2020). The Convention on Biological Diversity (CBD)’s Post-2020 target on invasive alien species–what should it include and how should it be monitored?. NeoBiota.

[CR31] Estévez RA, Anderson CB, Pizarro JC, Burgman MA (2015). Clarifying values, risk perceptions, and attitudes to resolve or avoid social conflicts in invasive species management.. Conserv Biol.

[CR32] Evans MC (2021). Re-conceptualizing the role(s) of science in biodiversity conservation.. Environ Conserv.

[CR33] Foxcroft LC, McGeoch M (2011). Implementing invasive species management in an adaptive management framework. Koedoe.

[CR34] Frumento PZ, Whitmer WE, Alter TR, Martin P, Alter TR, Hine D, Howard T (2019). Strategy and practice for community engagement. Community-based control of invasive species.

[CR35] Game ET, Fitzsimons JA, Lipsett-Moore G, McDonald-Madden E (2013). Subjective risk assessment for planning conservation projects.. Environ Res Lett.

[CR36] García-Díaz P, Cassey P, Norbury G (2021). Management policies for invasive alien species: addressing the impacts rather than the species.. BioScience.

[CR37] Green SJ, Grosholz ED (2021). Functional eradication as a framework for invasive species control.. Front Ecol Environ.

[CR38] Groves CR, Game ET (2016). Conservation planning: informed decisions for a healthier planet.

[CR39] Hawkins CL, Bacher S, Essl F (2015). Framework and guidelines for implementing the proposed IUCN Environmental Impact Classification for Alien Taxa (EICAT).. Diversity Distrib.

[CR40] Hemming V, Burgman MA, Hanea AM (2018). A practical guide to structured expert elicitation using the IDEA protocol.. Methods Ecol Evol.

[CR41] Hulme PE (2021). Unwelcome exchange: international trade as a direct and indirect driver of biological invasions worldwide. One Earth.

[CR42] Kapitza K, Zimmermann H, Martín-López B, von Wehrden H (2019). Research on the social perception of invasive species: a systematic literature review.. NeoBiota.

[CR43] Kumschick S, Bacher S, Dawson W (2012). A conceptual framework for prioritization of invasive alien species for management according to their impact.. NeoBiota.

[CR44] Kumschick S, Measey GJ, Vimercati G (2017). How repeatable is the Environmental Impact Classification of Alien Taxa (EICAT)? Comparing independent global impact assessments of amphibians.. Ecol Evol.

[CR45] Kumschick S, Bacher S, Bertolino S (2020). Appropriate uses of EICAT protocol, data and classifications.. NeoBiota.

[CR46] Lambin X, Burslem D, Caplat P (2020). CONTAIN: optimising the long-term management of invasive alien species using adaptive management.. NeoBiota.

[CR47] Lang DJ, Wiek A, Bergmann M (2012). Transdisciplinary research in sustainability science: practice, principles, and challenges.. Sustain Sci.

[CR48] Latombe G, Canavan S, Hirsch H (2019). A four-component classification of uncertainties in biological invasions: implications for management.. Ecosphere.

[CR49] Linders TE, Bekele K, Schaffner U (2020). The impact of invasive species on social-ecological systems: relating supply and use of selected provisioning ecosystem services.. Ecosyst Serv.

[CR50] Liu S, Sheppard A, Kriticos D, Cook D (2011). Incorporating uncertainty and social values in managing invasive alien species: a deliberative multi-criteria evaluation approach.. Biol Invasions.

[CR51] Lodge DM, Simonin PW, Burgiel SW (2016). Risk analysis and bioeconomics of invasive species to inform policy and management. Annu Rev Environ Resour.

[CR52] Madani K, Pierce TW, Mirchi A (2017). Serious games on environmental management.. Sustain Cities Soc.

[CR53] Martinez-Cillero R, Willcock S, Perez-Diaz A (2019). A practical tool for assessing ecosystem services enhancement and degradation associated with invasive alien species.. Ecol Evol.

[CR54] Mattor K, Betsill M, Huber-Stearns H (2014). Transdisciplinary research on environmental governance: a view from the inside.. Environ Sci Policy.

[CR55] McGeoch MA, Genovesi P, Bellingham PJ (2015). Prioritizing species, pathways, and sites to achieve conservation targets for biological invasion.. Biol Invasions.

[CR56] Milanović M, Knapp S, Pyšek P, Kühn I (2020). Linking traits of invasive plants with ecosystem services and disservices. Ecosyst Serv.

[CR57] Mill AC, Crowley SL, Lambin X (2020). The challenges of long-term invasive mammal management: lessons from the UK.. Mammal Rev.

[CR58] Milner-Gulland EJ, Shea K (2017). Embracing uncertainty in applied ecology.. J Appl Ecol.

[CR59] Nentwig W, Bacher S, Pyšek P (2016). The generic impact scoring system (GISS): a standardized tool to quantify the impacts of alien species.. Environ Monit Assess.

[CR60] Novoa A, Shackleton R, Canavan S (2018). A framework for engaging stakeholders on the management of alien species.. J Environ Manag.

[CR61] Nuñez MA, Chiuffo MC, Torres A (2017). Ecology and management of invasive Pinaceae around the world: progress and challenges.. Biol Invasions.

[CR62] Nuñez MA, Davis KT, Dimarco RD (2021). Should tree invasions be used in treeless ecosystems to mitigate climate change?. Front Ecol Environ.

[CR63] Nyumba OT, Wilson K, Derrick CJ, Mukherjee N (2018). The use of focus group discussion methodology: Insights from two decades of application in conservation.. Methods Ecol Evol.

[CR64] O’Connor RA, Nel JL, Roux DJ (2021). The role of environmental managers in knowledge co-production: insights from two case studies.. Environ Sci Policy.

[CR65] Oficialdegui FJ, Delibes-Mateos M, Green AJ (2020). Rigid laws and invasive species management.. Conserv Biol.

[CR66] Probert AF, Ward DF, Beggs JR (2020). Conceptual risk framework: integrating ecological risk of introduced species with recipient ecosystems.. BioScience.

[CR67] Pyšek P, Hulme PE, Simberloff D (2020). Scientists’ warning on invasive alien species.. Biol Rev.

[CR68] Ricciardi A, Hoopes MF, Marchetti MP, Lockwood JL (2013). Progress toward understanding the ecological impacts of nonnative species.. Ecol Monogr.

[CR69] Robertson PA, Mill A, Novoa A (2020). A proposed unified framework to describe the management of biological invasions.. Biol Invasions.

[CR70] Rose DC, Sutherland WJ, Amano T (2018). The major barriers to evidence‐informed conservation policy and possible solutions.. Conserv Lett.

[CR71] Rowland EL, Cross MS, Hartmann H (2014) Considering multiple futures: scenario planning to address uncertainty in natural resource conservation. US Fish and Wildlife Service, Washington

[CR72] Roy HE, Peyton J, Aldridge DC (2014). Horizon scanning for invasive alien species with the potential to threaten biodiversity in Great Britain. Glob Change Biol.

[CR73] Roy HE, Peyton JM, Booy O (2020). Guiding principles for utilizing social influence within expert-elicitation to inform conservation decision-making.. Glob Change Biol.

[CR74] Russell-Smith J, Lindenmayer D, Kubiszewski I (2015). Moving beyond evidence-free environmental policy. Front Ecol Environ.

[CR75] Samson E, Hirsch PE, Palmer SC (2017). Early engagement of stakeholders with individual-based modeling can inform research for improving invasive species management: the Round Goby as a sase study.. Front Ecol Evol.

[CR76] Seebens H, Bacher S, Blackburn TM (2021). Projecting the continental accumulation of alien species through to 2050. Glob Change Biol.

[CR77] Shackleton RT, Richardson DM, Shackleton CM (2019). Explaining people’s perceptions of invasive alien species: a conceptual framework.. J Environ Manag.

[CR78] Simberloff D (2003). How much information on population biology is needed to manage introduced species?. Conserv Biol.

[CR79] Sutherland WJ, Dicks LV, Everard M, Geneletti D (2018). Qualitative methods for ecologists and conservation scientists.. Methods Ecol Evol.

[CR80] Turbelin AJ, Malamud BD, Francis RA (2016). Mapping the global state of invasive alien species: patterns of invasion and policy responses. Glob Ecol Biogeogr.

[CR81] Van Woensel L (2019) A bias radar for responsible policy-making: foresight-based scientific advice. Springer Nature, Cham, Switzerland

[CR82] Vaz AS, Kueffer C, Kull CA (2017). The progress of interdisciplinarity in invasion science. Ambio.

[CR83] Vimercati G, Kumschick S, Probert AF (2020). The importance of assessing positive and beneficial impacts of alien species.. NeoBiota.

[CR84] Westgate MJ, Likens GE, Lindenmayer DB (2013). Adaptive management of biological systems: a review.. Biol Conserv.

[CR85] Woodford DJ, Richardson DM, MacIsaac HJ (2016). Confronting the wicked problem of managing biological invasions.. NeoBiota.

