# Supplementary material for: Identifying Priorities, Targets, and Actions for the Long-term Social and Ecological Management of Invasive Non-Native Species
Source: Environ Manage. 2021 Sep 29;69(1):140–53. doi: 10.1007/s00267-021-01541-3 (PMC8758626; doi:10.1007/s00267-021-01541-3)

**Online Resource 2.**

**Figure.** Formulating priorities, targets, and high-level actions for the long-term management of invasive non-native species. The first stage involves inventorying impact outcomes, impact mechanisms, impact magnitude and levels of confidence – our process involved open discussions (top) and collation in spreadsheets. In the second stage, those impact outcomes are prioritised for action – facilitated workshop for sharing ideas and information (top) and an example of prioritised outcomes and corresponding high-level actions. Once priority impact outcomes and high-level actions are agreed, quantitative indicators have to be proposed – examples include measuring invasive non-native species abundance (plant abundance – right) and population trends of impacted species (hooded grebe impacted by American mink predators; left). Photos: J. Cristóbal Pizarro (workshop), Priscila A. Powell (privet), and Ignacio González (hooded grebe; *Podiceps gallardoi*).


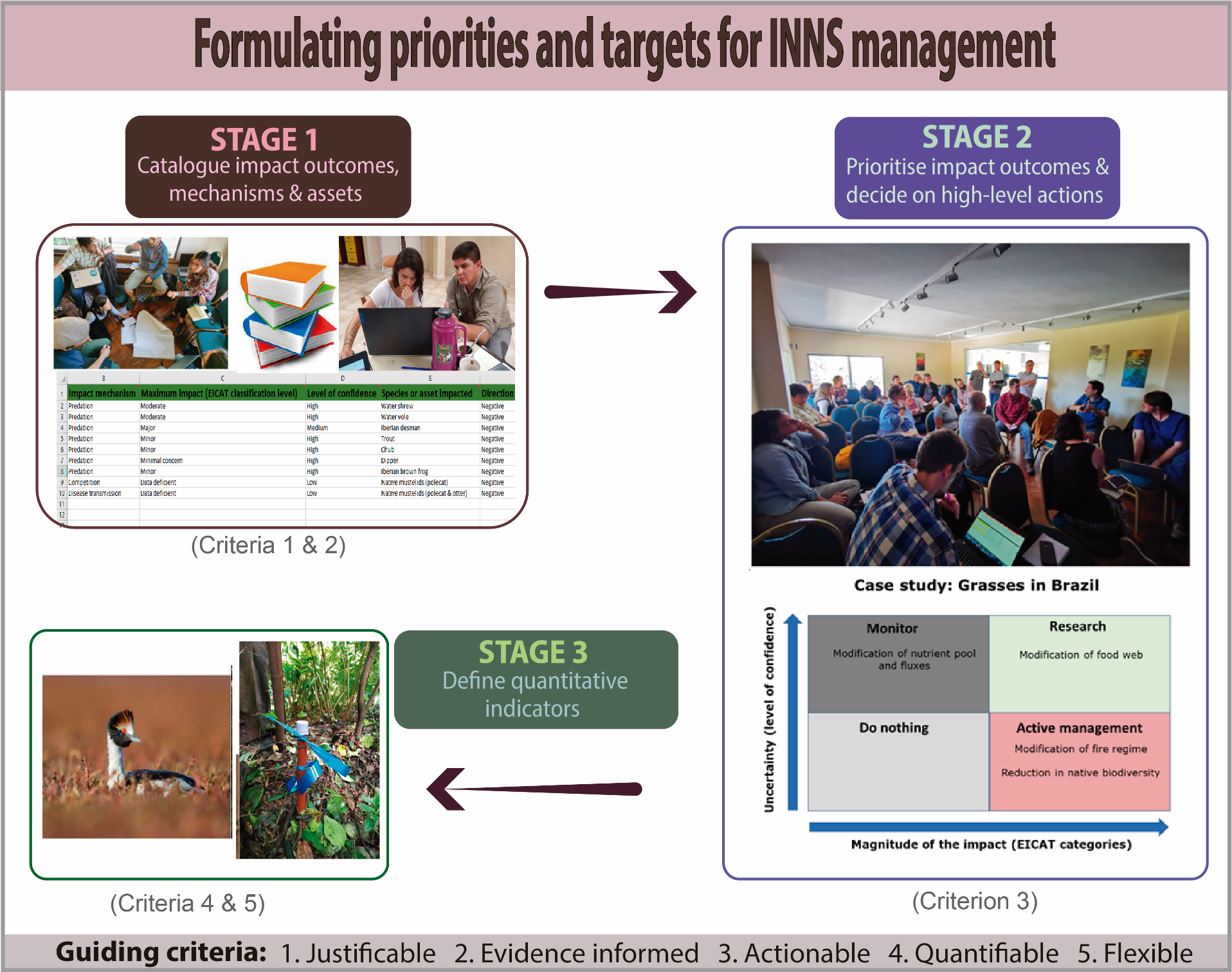

Supplement: Supplementary file 2 — ESM 2 [file 267_2021_1541_MOESM2_ESM.docx]
