# Supplementary material for: Identifying Priorities, Targets, and Actions for the Long-term Social and Ecological Management of Invasive Non-Native Species
Source: Environ Manage. 2021 Sep 29;69(1):140–53. doi: 10.1007/s00267-021-01541-3 (PMC8758626; doi:10.1007/s00267-021-01541-3)

**Online Resource 3. Figure.** The number of impact outcomes inventoried in each Impact Score spreadsheet was higher for INNS plants than for animals (top; 17 spreadsheets in total). This boxplot shows the median (black line), the interquartile range (box; 25% and 75% quartiles), and the maximum and minimum values (whiskers). The points are the actual values for comparison. The number of impact outcomes per spreadsheet had little relationship to the area (km^2^) or the amount of time that it took for experts to complete their Impact Score spreadsheets (bottom; 17 spreadsheets in total).


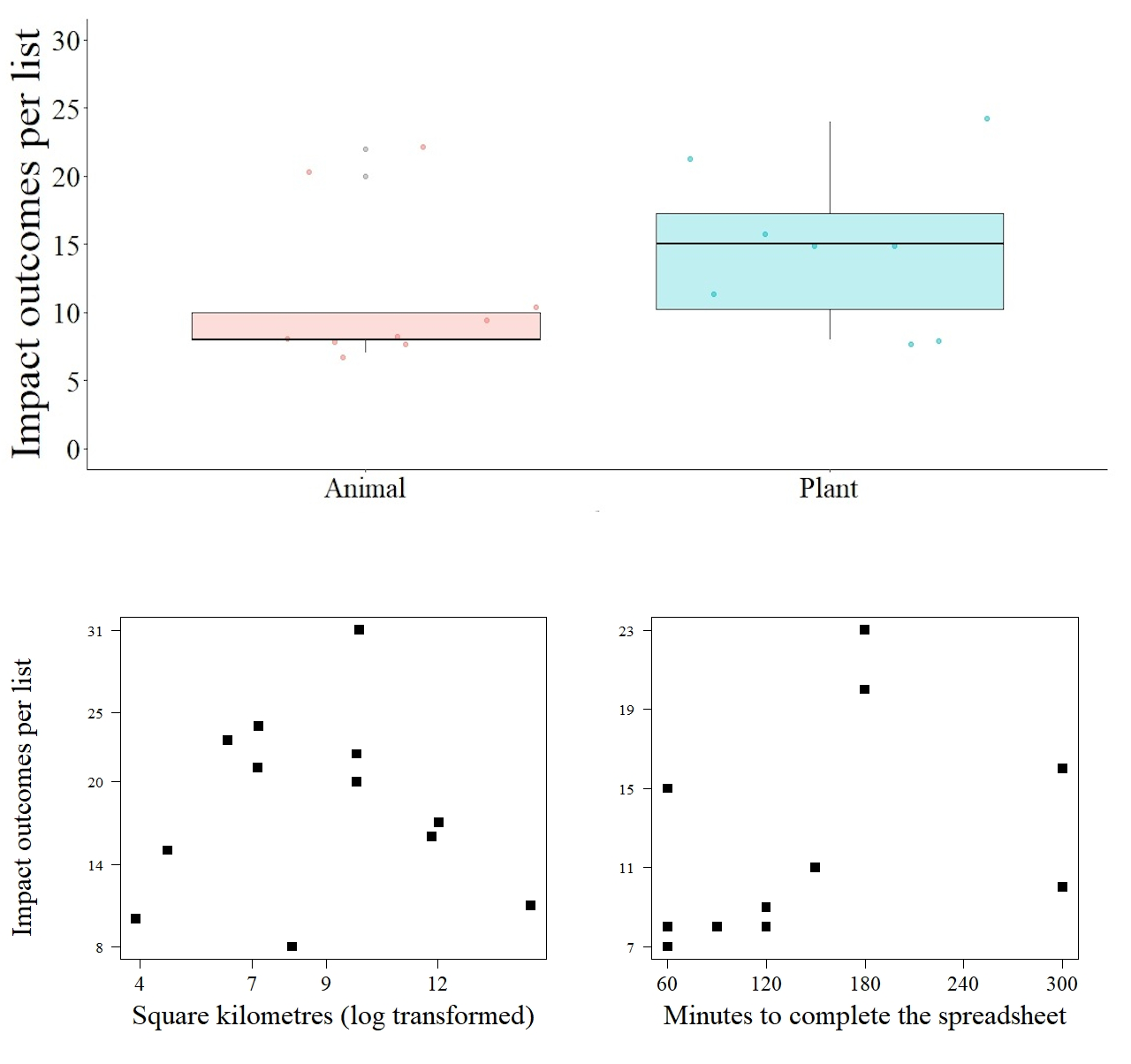

Supplement: Supplementary file 3 — ESM 3 [file 267_2021_1541_MOESM3_ESM.docx]
