# Supplementary material for: Identifying Priorities, Targets, and Actions for the Long-term Social and Ecological Management of Invasive Non-Native Species
Source: Environ Manage. 2021 Sep 29;69(1):140–53. doi: 10.1007/s00267-021-01541-3 (PMC8758626; doi:10.1007/s00267-021-01541-3)
Supplement: Supplementary file 4 — ESM 4 [file 267_2021_1541_MOESM4_ESM.docx]

**Online Resource 4. Figure.** The prevalence of impact outcomes evaluated as massive or major (Environmental Impact Classification of Alien Taxa - EICAT categories) for each of the seven case studies (top). Invasive non-native animals exhibited a higher percent of socioeconomic impact outcomes than invasive non-native plants (bottom; based on 17 spreadsheets). The bottom boxplot shows the median (black line), the interquartile range (box; 25% and 75% quartiles), and the maximum and minimum values (whiskers). The points are the actual values for comparison.

**
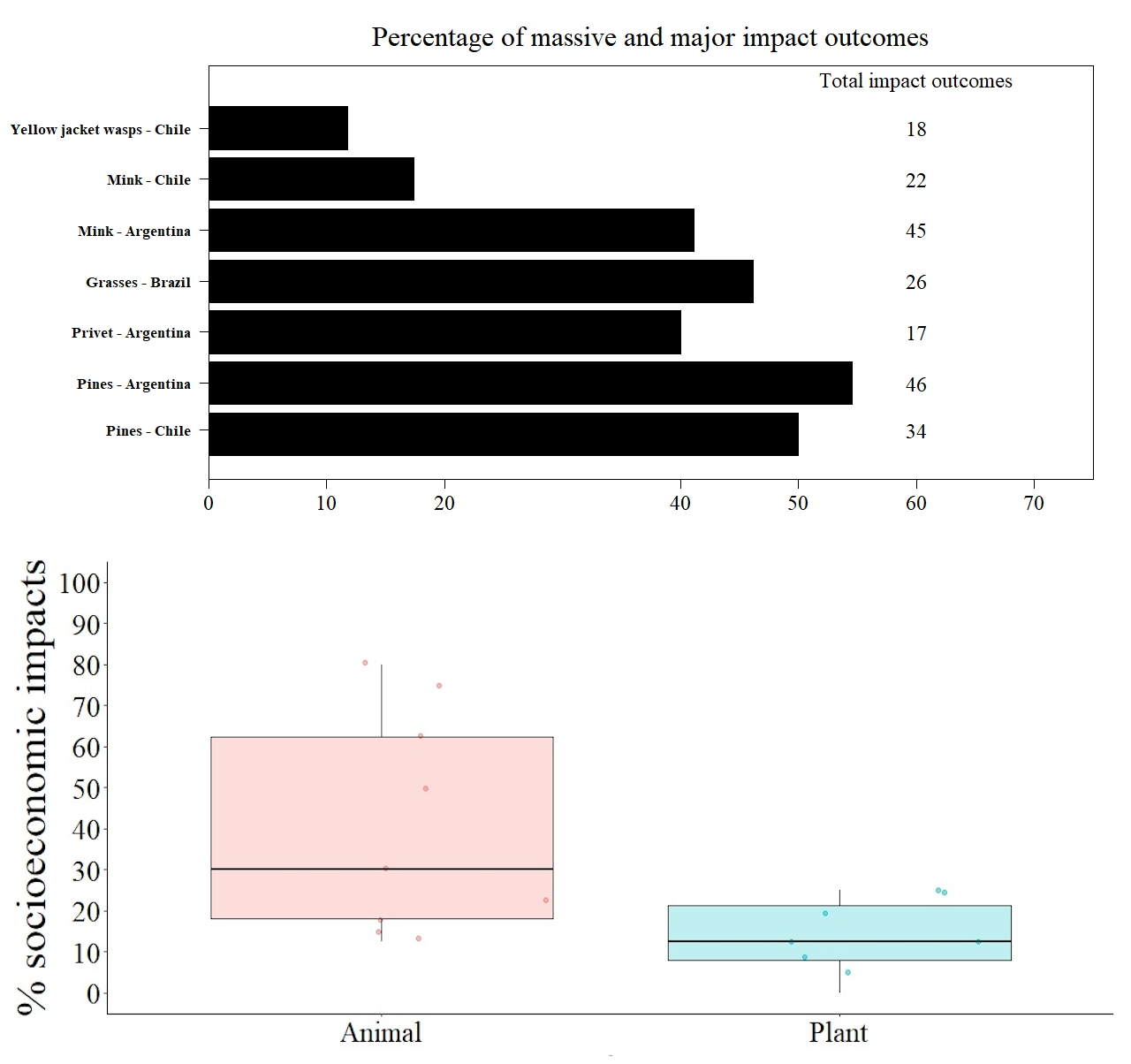
**
